# Remote activity monitoring for family caregivers of persons living with dementia: a mixed methods, randomized controlled evaluation

**DOI:** 10.1186/s12877-021-02634-8

**Published:** 2021-12-18

**Authors:** Joseph E. Gaugler, Rachel Zmora, Lauren L. Mitchell, Jessica Finlay, Christina E. Rosebush, Manka Nkimbeng, Zachary G. Baker, Elizabeth A. Albers, Colleen M. Peterson

**Affiliations:** 1 Center for Healthy Aging and Innovation, Division of Health Policy and Management, School of Public Health, University of Minnesota, D351 Mayo (MMC 729), 420 Delaware Street S.E, Minneapolis, MN 55455 USA; 2 Division of Epidemiology and Community Health, School of Public Health, University of Minnesota, Minneapolis, MN USA; 3 Department of Psychology, Emmanuel College, Boston, MA USA; 4 Social Environment and Health Program, Institute of Social Research, University of Michigan, Ann Arbor, MI USA

**Keywords:** Technology, Alzheimer’s disease, Aging in place, Community, Home, Caregiving

## Abstract

**Background:**

The goal of the present study was to determine whether a remote activity monitoring (RAM) system benefited caregivers who aided relatives with Alzheimer’s disease or related dementias (ADRD) living at home. We hypothesized that over 18 months, families randomly assigned to receive RAM technology in the home of the person with ADRD would experience statistically significant (*p* < .05): 1) improvements in caregiver self-efficacy and sense of competence when managing their relative’s dementia; and 2) reductions in caregiver distress (e.g., burden, role captivity, and depression).

**Methods:**

An embedded mixed methods design was utilized, where 179 dementia caregivers were randomly assigned to receive RAM or not. Caregivers were surveyed bi-annually over an 18-month period to collect quantitative and qualitative data on RAM’s effects. Semi-structured interviews with 30 caregivers were completed following the 18-month data collection period to explore more in-depth how and why RAM was perceived as helpful or not.

**Results:**

Growth curve models showed no direct or moderation effect of RAM on dementia caregiver outcomes. The qualitative data revealed a complex utilization process of RAM influenced by the care environment/context as well as the temporal progression of ADRD and the caregiving trajectory.

**Conclusions:**

The findings suggest the need for developing more effective mechanisms to match appropriate technologies with the heterogeneous needs and care contexts of people living with ADRD and their caregivers. A triadic approach that incorporates professional care management alongside passive monitoring systems such as RAM may also enhance potential benefits.

**Trial registration:**

ClinicalTrials.govNCT03665909, retrospectively registered on 11 Sept 2018.

**Supplementary Information:**

The online version contains supplementary material available at 10.1186/s12877-021-02634-8.

## Introduction

Unpaid caregivers, particularly family members, are the core of the long-term care system in the United States for people with Alzheimer’s disease or related dementias (ADRD). Approximately 10% of Americans over the age of 65 have ADRD, with this percentage escalating to 32% for those over the age of 85 [1]. Eighty-three percent of older persons with dementia rely solely on unpaid caregivers for needed assistance [2]. The multi-year progression of ADRD has placed considerable pressure on the U.S. healthcare system, as persons with dementia account for considerably higher healthcare costs than individuals without ADRD [1]. Complicating these issues is the impending shortage of family caregivers in the U.S., with the available caregiver to older person in need ratio anticipated to drop from 7:1 in 2010 to 4:1 in 2030 [3]. In addition a large, descriptive literature has indicated the increased risk for emotional distress, negative mental health, and other adverse outcomes for dementia caregivers when compared to caregivers of persons with other chronic conditions or age-matched non-caregivers (see [1]). Although evidence is mixed, intervention studies that focus on building self-competence/efficacy when managing the functional, cognitive, and behavioral care demands of persons with dementia may hold promise when alleviating stress and negative mental health outcomes among caregivers [1].

Among the options to offset the impending family care gap in the U.S. is technology [4, 5]. Technology interventions for older persons are generally utilized as real-time data capture tools to complement clinical or family care for older persons or as interventions themselves designed to improve important health outcomes. Although research on novel technological interventions for people with ADRD and their family caregivers has grown considerably over the past two decades, much of this work continues to focus on design, feasibility, and acceptability (with a need for conceptual refinement in these areas) [6, 7] and less on controlled outcome studies that ascertain whether these technologies benefit the well-being of persons with ADRD and their caregivers [8–10]. The objective of this experimental mixed methods demonstration was to determine the 18-month effectiveness of remote activity monitoring (RAM) technology in improving outcomes among family caregivers or community-dwelling persons with ADRD.

### Background

Several qualitative and feasibility studies have examined the potential utility and acceptability of various technologies to enhance care for people with ADRD, including robots [9, 11], wearable devices and home-based monitoring systems [12], augmented and mixed reality, and many others [4, 5, 13, 14]. Technology reviews have categorized various dementia care technologies as having seven broad functions: memory support, treatment, safety, security (most common), training, care delivery, social interaction, and other functions [15]. Dementia care technologies are targeted to people with ADRD across the spectrum of disease severity and to both family caregivers and professional care staff. In general, the science of dementia care technology has not focused on real-world application of technologies, often leaving caregivers and others to “repurpose” existing technologies to enhance their utility [15, 16].

Qualitative research examining preferences related to remote monitoring technology found that adult child caregivers were generally more likely to prefer remote technologies when compared to their mothers who were care recipients and overestimated their mothers’ comfort with remote activity monitoring (RAM) technology. In general, privacy was indicated as a significant concern among both adult children and their mothers. In addition, caregivers of people with ADRD often did not know “where to start” when it came to selecting assistive technologies, and healthcare providers offered little guidance [16, 17]. Other reviews of stakeholder perspectives of technology in dementia care have emphasized the importance of ease of use and flexibility, safety, privacy, and confidentiality as important issues to consider when using technologies to support care for persons with ADRD [18, 19]. Our prior mixed methods study of the acceptability and feasibility of RAM for dementia caregivers found that although perceived as appropriate for and useful to supplementing at-home caregiving, considerable customization of the system was needed to meet the needs of the person with ADRD as well as their family caregivers [20].

A handful of controlled studies have evaluated the efficacy or effectiveness of RAM for dementia caregivers. Existing studies tend to feature smaller samples (*n* < 60) and shorter follow-up (e.g., 12 months or less), but have indicated potentially positive effects of RAM technology use such as reduced distress on the part of dementia caregivers [21–24]. A larger scale, controlled evaluation of RAM found reductions in users’ healthcare costs, although the differences were not statistically significant [25]. In a preliminary 6-month study of RAM effectiveness, we found that RAM was not associated with any change in dementia caregiver competence, self-efficacy, or distress [26]. However, qualitative data indicated several potential moderators of efficacy, and in post-hoc empirical analyses we found that dementia caregivers who utilized RAM for relatives with ADRD who were less cognitively impaired and had difficulty navigating the home were more likely to indicate increased competence and self-efficacy over a 6-month period. The results also indicated that the initial months of calibrating the RAM system was critical to ensure benefit for dementia caregivers. Although the evidence base of effectiveness and efficacy of various technologies to support dementia caregivers has expanded considerably, how these interventions are implemented into everyday clinical or at-home care contexts over time requires greater conceptual and methodological refinement [27].

The goal of the present study was to advance understanding of whether a RAM system benefited caregivers who aided relatives with ADRD who lived at home. Although technologies such as RAM are often marketed as effective in allowing people with dementia to remain at home and to improve feelings of security and well-being on the part of dementia caregivers, rigorous, controlled research addressing these postulations remains relatively rare. Specifically, RAM technology provides the opportunity for more continuous monitoring of behaviors and activities of persons with dementia, thus allowing dementia caregivers a potentially greater sense of security as well as competence when managing care demands. Moreover, the design of RAM to issue alerts that may predict a subsequent adverse health event (e.g., fall, wandering) positions this technology as more proactive than reactive, which could also drive reductions in stress on the part of dementia caregivers. To this end, we hypothesized that over 18 months families randomly assigned to receive RAM technology in the home of the person with ADRD would experience statistically significant (*p* < .05): 1) improvements in caregiver self-efficacy and sense of competence when managing their relative’s ADRD, and 2) reductions in caregiver distress (e.g., burden, role captivity, and depression). Based on preliminary 6-month findings [26], we further examined how the context of at-home dementia care moderated the effects of RAM technology using longitudinal qualitative data collected throughout the demonstration evaluation.

The use and integration of the qualitative data during and following the collection of quantitative data was designed to yield greater insights as to the mechanisms of RAM benefit or lack thereof (i.e., the “how and why” RAM is effective or not) in contrast to solely relying on the longitudinal quantitative data to address the hypotheses of interest. For these reasons, we included a research question to further take advantage of the qualitative data available: What changed over time that influenced perceptions, engagement, and ultimately outcomes among dementia caregivers who used RAM?

## Methods

This study adheres to CONSORT (http://www.consort-statement.org/) guidelines.

### Conceptual model

**Fig. 1:**
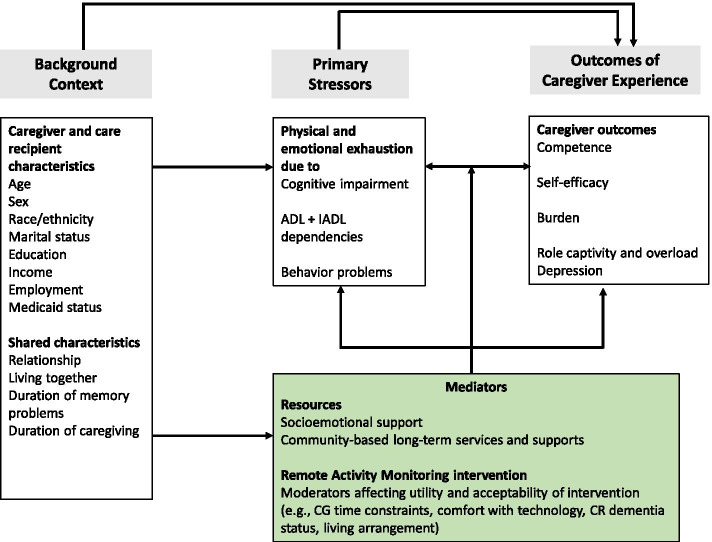
Framework for Understanding effectiveness of Remote Activity Monitoring on Caregiver and Care Recipient Outcomes. Note: CG = caregiver; CR = care recipient; ADL = activities of daily living; IADL = instrumental activities of daily living

For the present study, we adapted the Stress Process Model (SPM) to assess the effects of RAM technology on caregiver health outcomes (Fig. 1). The SPM posits that the physical and emotional stress of caregiving (primary stressors) proliferates to other life domains which then adversely influence global caregiving outcomes [28]. Previous applications of the SPM have evaluated the effects of resources, including various technology and non-technological interventions, on the proliferation of stress [29–31]. We frame RAM technology as a resource and consider covariates from three SPM domains: context of care (caregiver and care recipient sociodemographic characteristics), primary stressors (objective measures of ADRD severity), and resources (socioemotional support and use of community-based long-term services and supports).

### Design

**Fig. 2:**
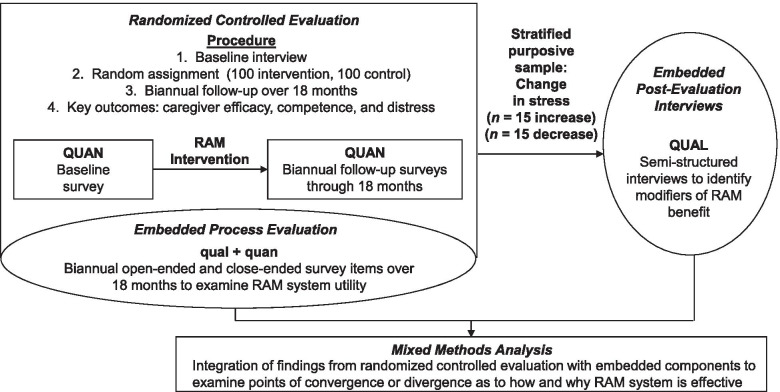
Embedded Experimental Mixed Methods Design. *Note.* RAM = Remote Activity Monitoring; ADRD = Alzheimer’s disease and related dementia; QUAN = quantitative strand, prioritized; QUAL = qualitative strand, prioritized

We used an embedded experimental mixed methods design, collecting qualitative data within the structure of a traditional randomized controlled evaluation ([QUAN+qual] → QUAL) [32]. We embedded qualitative data collection to examine and refine the process of RAM implementation during the evaluation and to determine why RAM technology was or was not effective for ADRD caregivers following completion of the trial (Fig. 2). An embedded experimental design was selected because it allowed for the opportunity to not only understand *whether* RAM was effective in addressing various dementia caregiving outcomes, but also *why/how* RAM was effective (or ineffective) doing so. By integrating the qualitative components during RAM use and following completion of the randomized controlled trial, our embedded experimental mixed methods evaluation yielded more insights as to the mechanisms of benefit or lack thereof.

### Remote activity monitoring system

The RAM system evaluated in the current study has been described in detail previously [20, 26, 33]. Briefly, the RAM system consists of motion sensors that are placed throughout the home of the person with ADRD (e.g., front door, bed, refrigerator door, toilet). The sensors operate jointly to monitor the person’s daily activity. For the study, a Director of Health Technology (DHT) conducted an initial needs assessment with each caregiver and tailored sensor placement and configuration accordingly. Following installation, sensor data were collected and analyzed using algorithms to determine a baseline activity pattern for each care recipient.

The RAM system alerted caregivers to abnormal activity patterns via phone or e-mail. Caregivers could specify the activity patterns for which they wanted to receive alerts (e.g., care recipient getting out of bed at night or remaining in the bathroom too long). Caregivers were also alerted if the care recipient used an emergency call necklace that was provided along with the RAM system. Finally, caregivers were given access to a web-based dashboard that provided scheduled reports summarizing sensor activity. Caregivers were encouraged to share summary data with their care recipient’s health care provider.

### Sample and procedures

We recruited community-dwelling caregiver/care recipient dyads between July 2014 and September 2018 using the University of Minnesota Caregiver Registry (comprised of ADRD caregivers who voluntarily agreed to be contacted for future studies following outreach and educational events), advertisements, community presentations, and referrals from professionals. Follow-up data collection continued until April 2020 (when the final scheduled survey was completed and returned). Research coordinators contacted potentially eligible caregivers to explain the study and screen those who were interested for eligibility. Caregivers were required to be English-speaking, ≥21 years of age, self-identify as the primary caregiver, and plan to remain in the area for ≥18 months. Care recipients were required to be English-speaking, have a physician diagnosis of ADRD, be ≥55 years of age, and not receive case management services.

Coordinators administered consent in-person or via phone, and signed consent was obtained from all participating caregivers. Care recipients were asked to complete a cognitive screening (the St. Louis University Mental Status [SLUMS] examination) and provide assent. Written assent was obtained for care recipients who scored ≥20 on SLUMS; verbal assent was obtained for those who scored < 20. When applicable, signed consent was also obtained from the care recipient’s legally authorized representative.

Following consent and caregiver completion of a baseline survey, dyads were randomly assigned to receive the RAM system or an attention control (i.e., monthly telephone check-ins with research staff). The senior author randomized participants in a 1:1 fashion using http://randomizer.org; allocation was based on a priori assignment number. Random assignment was not concealed, nor was subsequent data collection blinded (see the Discussion section). The DHT contacted dyads assigned to the intervention within 2 weeks of randomization to initiate RAM consultation and installation.

Follow-up surveys were administered to caregivers in-person, online, or via phone or mail at 6, 12, and 18 months. To ensure ongoing engagement, research staff completed a phone check-in with all intervention and control caregivers at the time each survey was administered. Staff completed a participant disposition form detailing bereavement or care recipient transition to long-term care prior to the 6-, 12-, and 18-month survey intervals.

Approximately 1 year into the study, staff discovered that RAM system malfunction was frequently not reported to the DHT. In response, an additional monthly phone check-in was initiated for caregivers in the intervention arm. As noted in our prior research of the RAM system [20], it often took several months for families to become acclimated and comfortable with using the system and the alerts it generated. As dementia family caregivers utilized the system more frequently (or attempted to) in the ensuing months of the study, reports of issues with lack of alerts and other challenges became more common during our research staff members’ monthly check-in calls.

Staff obtained verbal assent from intervention care recipients every 6 months. In instances where the care recipient expressed a desire for removal, the DHT removed the RAM system from the home. Consistent with intention to treat, caregivers were asked to complete follow-up surveys regardless of RAM system placement. At the conclusion of the study, 30 caregivers from the intervention group were invited to participate in semi-structured phone interviews (15 who indicated high RAM acceptance and 15 who indicated low RAM acceptance on the RAM system review checklist; see below) to provide further insight as to why they felt the system did or did not work for them. All experimental protocols were approved by the University of Minnesota Institutional Review Board; the methods were carried out in accordance with relevant guidelines and regulations. Informed consent and/or assent was obtained from all participants. The protocol was retrospectively registered on ClinicalTrials.gov (NCT03665909) on September 11th, 2018.

### Measures

#### Demographics and context of care

Background and contextual factors collected included caregiver and care recipient demographics, relationship between caregiver and care recipient, living situation, and duration of memory loss and caregiving relationship (see Table 1, below).

**Table 1:** Baseline demographics of sample

Characteristic	Caregiver Intervention	Caregiver Control	*p*-value	Care Recipient Intervention	Care Recipient Control	*p*-value
Age, M (SD)	62.4 (10.8)	63.0 (12.5)	.696	78.2 (9.5)	78.4 (9.3)	.880
Female, N (%)	68 (77.3%)	73 (80.2%)	.561	50 (56.8%)	46 (50.6%)	.387
White, N (%)	85 (96.6%)	89 (97.8%)	.623	80 (90.9%)	86 (94.5%)	.354
Non-Hispanic, N (%)	88 (100%)	86 (94.5%)	.083	86 (97.7%)	86 (94.5%)	.526
Married, N (%)	70 (79.6%)	79 (86.8%)	.193	53 (60.2%)	56 (61.5%)	.857
Children, M (SD)	2.0 (1.6)	2.5 (2.0)	.088	2.8 (2.0)	3.2 (2.0)	.164
Graduated college, N (%)	59 (67.1%)	58 (63.7%)	.642	42 (47.7%)	44 (48.4%)	.933
Household income > 60 k, N (%)	50 (56.8%)	57 (62.6%)	.427	22 (25.0%)	35 (38.5%)	.053
Employment, N (%)						
Fulltime	26 (29.6%)	26 (28.6%)	.524			
Retired	32 (36.4%)	40 (44.0%)				
Other	30 (34.1%)	25 (27.5%)				
Relationship, N (%)						
Spouse	42 (47.7%)	50 (55.0%)				
Parent	39 (44.3%)	37 (40.7%)				
Other	7 (8.0%)	4 (4.4%)	.468			
Living arrangement, N (%)						
Alone				17 (19.3%)	24 (26.4%)	
With caregiver				52 (59.1%)	55 (60.4%)	
With another relative				7 (8.0%)	4 (4.4%)	
Other				12 (13.6%)	8 (8.8%)	.416
Medicaid, N (%)				16 (18.2%)	17 (18.7%)	.594
ADL, M (SD)	0.4 (1.2)	0.5 (1.4)	.650	2.1 (2.2)	2.4 (2.7)	.372
IADL, M (SD)	2.0 (3.8)	1.8 (3.4)	.725	8.2 (3.1)	7.9 (3.5)	.577
CI, M (SD)				13.7 (6.1)	12.6 (7.0)	.281
RMBPC-Frequency, M (SD)				35.9 (12.4)	32.5 (12.5)	.068
RMBPC-Reaction, M (SD)				19.3 (13.1)	19.3 (11.6)	.991

*ADL* activities of daily living, *IADL* instrumental activities of daily living, *CI* cognitive impairment, *RMBPC* revised memory and behavior problems checklist

#### Primary objective stressors

Primary objective stressors captured the nature and extent of the care recipient’s needs [28]. These included the level of assistance (no help, some help, or a lot of help) the care recipient required for six activities of daily living (ADLs) (α = .89 [34]; and six instrumental activities of daily living (IADLs) (α = .96 [35];. Eight items were used to measure level of care recipient cognitive impairment (e.g., ‘how difficult is it for the person with memory loss to know what day of the week it is?’) on a 5-point scale ranging from ‘can’t do it at all’ to ‘not at all difficult’ (α = .86 [28];. The Revised-Memory and Behavior Problems Checklist was used to measure frequency of 30 common dementia-related behaviors (α = .89 [36];.

#### Resources

An 8-item measure of socioemotional support assessed the help provided to the caregiver by family and friends on a 5-point scale (1 = ‘strongly disagree’ to 5 = ‘strongly agree;’ α = .68) [28]. A checklist of community-based long-term services and supports (LTSS), developed by the authors, further queried the caregiver’s use of paid services (e.g., housekeeping, transportation, respite programs) to support their care recipient. Finally, caregivers were asked if their care recipient currently used any monitoring technology apart from the RAM system such as an emergency call pendant or bracelet.

#### Caregiver self-efficacy and sense of competence

Self-efficacy was measured by asking caregivers to rate their confidence in achieving eight caregiving tasks on a 5-point scale ranging from ‘very unconfident’ to ‘very confident’ (e.g., ‘do something to keep the person with memory loss as independent as possible’) (α = .86) [37]. The Short Sense of Competence Questionnaire (SSCQ) was used to measure caregiver competence for seven items (e.g., ‘I feel stressed between trying to give to the person with memory loss as well as meet my other responsibilities’) on a 5-point scale ranging from ‘strongly disagree’ to ‘strongly agree’ (α = .74 [38];.

#### Caregiver distress

The Zarit Burden Interview was used to measure caregiver distress for 22 items (e.g., ‘do you feel the person with memory loss asks for more help than he/she needs?’) on a 5-point scale ranging from ‘never’ to ‘nearly always’ (α = .93 [39, 40];. Additionally, three items each were used to measure caregiving role captivity (α = .80) and overload (α = .81) (e.g., ‘how often do you wish you were free to lead a life of your own?’) on a 4-point scale ranging from ‘not at all’ to ‘very much’. Finally, depressive symptoms were measured using the 20-item Center for Epidemiological Studies-Depression scale (e.g., ‘during the past week, I was bothered by things that don’t usually bother me;’ on a 4-point response scale ranging from ‘rarely or none of the time’ to ‘most of the time;’ α = .94) [41].

#### RAM system review checklist

Caregivers in the intervention arm were asked to complete a 22-item review checklist rating the acceptability and utility of the RAM system on a Likert-type scale at 6, 12, and 18 months. Example items include, “The alerts generated by the [*RAM system*] have helped prevent crises for the person with memory loss” and “I would recommend the [*RAM system*] to others in a similar situation as the person with memory loss” (on a 5-point scale range from 1 = ‘strongly disagree’ to 5 = ‘strongly agree’). Caregivers were also asked eight open-ended questions about difficulty/ease of use, utility of system alerts and the web-based dashboard, and perceived effects of the RAM system on their caregiving role.

#### Semi-structured interviews

Caregivers in the intervention arm who were administered semi-structured interviews at the conclusion of the study were asked to provide further detail on why they felt the RAM system did or did not reduce caregiving distress or help manage their care recipient’s daily function. They were also asked to provide feedback on the benefits and drawbacks of the system. All interviews were digitally recorded and professionally transcribed. The qualitative interview guide is included in the Online Supplemental Material.

### Data analysis

#### Qualitative analysis

We analyzed open-ended responses from the 6-, 12-, and 18-month follow-up surveys as well as transcripts from the semi-structured interviews. All qualitative data were organized in the software package NVivo (Version 12).

We thematically analyzed the data using Braun and Clarke’s [42] six steps of thematic analysis: (1) familiarization; (2) generation of initial codes; (3) search for themes; (4) review themes; (5) define and name themes; and (6) write up themes. All authors read the data to familiarize and gain a sense of the whole. This supported immersion in the data to enable new insights to emerge and inductively develop categories without imposing preconceived categories [43]. After generating initial codes, authors JF, MN, LM, CR, CP, ZB, and EA compared interpretations and points of divergence to refine and clarify codes. We independently coded a sample of responses to check for consistency in meaning and application of the codebook and illuminate any differing interpretations via individual bias. After team discussions to finalize the codebook, authors JF, MN, LM, CP, ZB, and EA each coded a portion of the qualitative data. We reviewed each other’s coding to ensure completeness and accuracy and add any additional coding. Peer debriefing, referential adequacy, negative case analysis, and clear audit trails enhanced transparency and credibility [44]. Iterative analyses continually seeking interpretation, alternative understandings and linkages led to saturation, whereby the themes were well-described by and fitting with the data [45].

Peer debriefing occurred as experts in the field of dementia care met for extensive discourse to ensure alignment of understanding of themes. We employed referential adequacy by having coders each explore two interviews, which we used to create our initial set of themes. Coders were then assigned a distinct subset of interviews to apply those codes to.

Negative case analysis occurred through the revision of codes and themes based on disconfirming data, upon discovery of disconfirming data through consensus discussions among coders. We likewise updated our codebook to explicitly indicate when individual contexts were inappropriate for the use of a given code. Audit trails were created through the maintenance of extensive records including specific coders, how codes were applied, why a code was applied, and when it was applied, as well as when and how themes were newly created or modified to fit observations in the semi-structured interviews.

#### Quantitative and mixed methods analysis

To determine whether use of the RAM system improved caregiver outcomes we used latent growth curve modeling. We tested intercept-only, linear, and quadratic unconditional models. We used fit statistics including Akaike Information Criterion (AIC), Bayesian Information Criteria (BIC) and root mean square error of approximation (RMSEA) to identify the best functional form. We created models for each of the six primary outcomes: competence, self-efficacy, burden, role captivity and overload, and depression. Based on available statistical power calculators for growth curve models (http://saspower.psychstat.org) that consider both sample size and the number of data points available (*n* = 4), our analyses were powered to detect moderate to large changes in our selected outcomes.

Next, we added covariates to the best fitting unconditional model for each outcome. We modeled treatment (RAM system versus control), demographics and context of care variables as time-invariant covariates. Primary stressors identified in our preliminary 6-month effectiveness analysis were modeled as time-varying covariates. Using the results of the qualitative analysis (see Results), we mapped themes onto items measured in the survey where available. We modeled these items as dichotomous, time-varying covariates and added interaction terms for each covariate crossed with treatment. We adjusted for multiple tests using the Holm correction [46].

## Results

### Sample characteristics

Caregivers were 62.7 (SD = 11.7) years old on average at the time of enrollment. Close to 80 % (78.8%) of caregivers were women, 97.2% were non-Hispanic white, and 83.2% were married or living with a partner. Slightly over half (51.4%) of caregivers were the spouse or partner of the person with ADRD, and 42.5% were the daughter or son. Care recipients were 78.3 (SD = 9.3) years old on average. Over half of caregivers (53.6%) were women, 92.7% white, and 96.1% were non-Hispanic. Care recipients had a variety of living arrangements including at home alone (22.9%), with their caregiver (59.8%), and with another relative (6.1%) (Table 1). Caregivers and care recipients in the intervention and control groups were comparable at baseline.

**Fig. 3:**
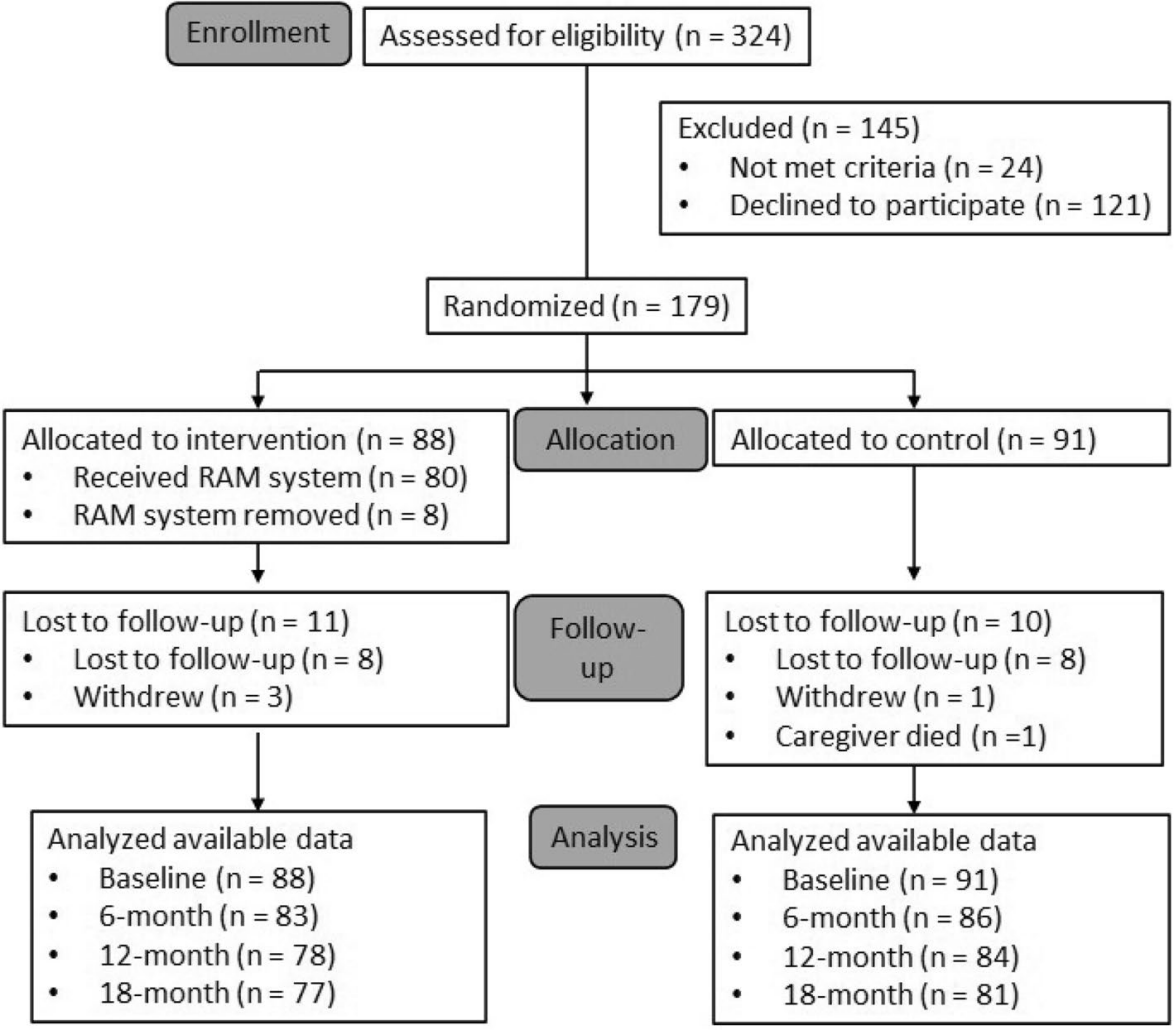
CONSORT diagram

Eighty-eight dyads were randomized to receive the RAM and 91 were randomized to attention control (Fig. 3). Of the 88 dyads engaged with the RAM at baseline, eight had the system removed early due to caregiver request or the care recipient not providing ongoing assent. Eleven intervention dyads were lost to follow-up, including three caregivers who requested to withdraw and eight who stopped returning surveys. Of the 91 dyads in the control group, 10 were lost to follow-up, including one caregiver who passed away, one who requested to withdraw, and eight who stopped returning surveys. Four care recipients in the treatment group and three in the control group passed away, though abbreviated follow-up surveys were still administered to their caregivers. Consistent with an intent-to-treat approach, available data from all treatment and control dyads were included in analyses.

The RAM system review checklist, administered separately to the treatment group, was completed by a subset of caregivers (66, 51, and 42 caregivers at 6, 12, and 18 months, respectively). Mean checklist scores were consistent over time (6-month mean = 3.76 [sd = 0.80], 12-month mean = 3.75 [sd = 0.98], 18-month mean = 3.76 [sd = 0.72]), indicating mild to moderate agreement that the RAM system was acceptable and useful. Scores suggested that the set-up process, including the initial needs assessment and information provided by the DHT on how to use the system, was acceptable or highly acceptable. At 18 months, scores indicated agreement that the alerts were helpful (mean = 4.23 [sd = 1.19]) and that caregivers would recommend the system to others in a similar situation to themselves (mean = 4.00 [sd = 1.10]) or their care recipient (mean = 4.07 [sd = 1.00]. Caregivers also indicated agreement that the system helped prevent crises for the care recipient (mean = 3.74 [sd = 1.37]). Additional results on the frequency of sensor alerts and caregiver use of the RAM system have been published previously (Zmora et al., [33]).

### Qualitative findings

The qualitative analysis identified seven themes that reflected dementia caregivers’ changing perceptions of and engagement with the RAM system over time as well as reasons why the RAM system was perceived as beneficial or not. The themes, described below, reflect time-varying factors regarding caregiver and care recipient needs, preferences, and perspectives as well as fluid life contexts (all names of participants are changed to protect confidentiality).

#### Adjustment period

Caregivers and their surrounding support networks varied in their ability to customize the RAM system so that it suited their needs over time. Douglas (54 years old [y], male [M], 18 month [m] interview) recounted his initial high stress levels: “I kept getting calls and calls, and I’m going ‘which button do I hit?’ And then once we figured it out… I didn’t hear nothing for a while.” Douglas continued that he was able to relax after learning which buttons to press and reconfiguring the notification system for no calls from midnight to 6 am: “cause I needed my sleep too. And then they put [notifications] on an email to me.” Douglas’s perceptions of the system improved after a troubled start.

Multiple participants discussed the learning-intensive initial period with the RAM system. Dawn (57y, female (F), 6 m checklist) shared that “some of the charts were difficult to use at first but I learned how to read them.” Maria (61y, F, 6 m checklist) noted: “I have learned how to use [the RAM system] more effectively throughout the past six months with excellent training (emails, phone calls, webinars) from [the DHT]. It’s easy to use (if you have some familiarity with technology)... Initially there seems to be so much information but you learn.” Other participants moved sensors in response to false alarms and troubleshooted issues themselves. In contrast, Valerie (71y, F, 18 m interview) shared: “I tried and it’s like ‘oh this is too confusing, I’m lost’. It’s not user friendly.” Janice (51y, F, 12 m checklist) similarly noted that “I tried to use it in the beginning but found it too difficult so I gave up.... I felt like I should have been able to figure it out and when I couldn’t, I stopped asking for help.”

#### Level of ongoing technological support

Participants varied over the follow-up period in their level of engagement and assistance from the RAM’s DHT. Kathleen (54y, F, 6 m checklist) shared the following:

DHT interactions were always positive and helpful- from troubleshooting to serving as a liaison when we first started with the study. Her overall knowledge of the living situation, and the wisdom added as I worked out a notification plan, meant that other than building-specific challenges, participation ran smoothly.

These experiences and ongoing support contributed to high system rating reviews from Kathleen (18 m interview):

[The DHT] was there when you needed her to be there. She would check in periodically and—because I would check in with her. There were times she didn’t need to check in with me cause she knew exactly where we were. So I just remember checking in with her only a couple of times, but it was well-timed, and if I needed to get a hold of her, she was there.

The DHT provided support and check-ins throughout the process, as well as reacting to acute instances such as replacing monitors or fixing system malfunctions. Joyce (56y, F, 12 m checklist) noted the DHT’s patience, helpfulness, compassion, and availability to answer questions and make system adjustments during multiple interactions.

Not all participants had positive DHT experiences or assistance across the entire follow-up period. Janice (51y, F, 18 m checklist) noted that “[The DHT] was great but I think had a lot on her plate making it difficult for follow-up.” Peg (74y, F, 18 m checklist) similarly expressed: “[The DHT was] very helpful initially. Service seemed to wane a bit as time passed.” These experiences contributed to diminishing expectations and use of the RAM. Donna (64y, F, 6 m checklist) noted: “Training too short. No check-ins.” Julie (50y, F, 18 m checklist) relayed: “We have never gotten an alert. Could use a refresher course perhaps to make sure we are properly utilizing all the [RAM] can do for us.” Participants expressed the need for ongoing training and refresher courses to continue using the system beyond the startup period.

#### Care recipient reaction over time

Some care recipients experienced changing reactions to RAM sensors over the follow-up period. Both positive and negative changes in attitudes toward the sensors were reported. For example, Debra (54y, F, 6 m checklist) stated that her husband “doesn’t really want to be monitored...I get the impression, he can’t wait till [the sensors] are gone!!” However, when the same participant was asked about her husband’s perceptions of the sensors in the semi-structured interview, she reported, “I think he quickly forgot about them.” These statements suggest that the care recipient’s initial wariness of the sensors wore off over time, until he no longer paid attention to them. In contrast, Angela (67y, F, 18 m interview) reported that her care recipient eventually became increasingly frustrated with the system over time: “After a while [CR] just saw it as an intrusion, you know, that they were watching him constantly, and why do they have to know how many times I go to the bathroom? And why do they have to know when I open the refrigerator door?” Rather than acclimating to the system, this care recipient found it more intrusive as time went on.

#### ADRD progression

Caregivers discussed how the progression of care recipients’ ADRD influenced which features of the RAM system were or were not beneficial over time. Maria (61y, F, 12 m checklist) mentioned that “the alerts are the most useful tool in the [RAM] system for me right now because my mother’s Alzheimer’s has advanced so that I have to be at her home every day… The day-to-day monitoring from my computer isn’t as crucial but the alerts let me know about the situations of concern.” Theresa (56y, F, 12 m checklist) concurred that the alerts were vital as the disease increased in severity, sharing “with dad, progressively, the alerts are more important than ever.”

As care recipients’ ADRD progressed, some caregivers found that the RAM system was modifiable to better fit their caregiving needs over time. Debra (54y, F, 18 m interview) said that “because things changed for me and for [CR], … we don’t need to have all these things in place. Especially the refrigerator [sensor] and things like that… I said the front door [sensor], I’d like to keep that one.” However, not all caregivers had time available to change the RAM system to better meet their needs or even learn how the system worked. Heidi (67y, F, 18 m interview) also shared how the RAM system was not beneficial as her care recipient’s ADRD progressed. “His disease progressed in a way where that wasn’t particularly helpful. He’s not a wanderer. He still has really good judgment. He’s declined, but in ways different than I anticipated when I enrolled [in the study].”

#### Medical insights and tracking

Participants reported bringing the RAM data to physicians and other medical professionals as evidence of their care recipient’s medical needs. For example, Randy (74y, M, 18 m interview) showed how the care recipient was not sleeping in order to start a care plan: “To be able to have some discussion with a physician about, you know, lack of sleep.... We had something to back up our request for, let’s do something about this business of sleeping.” Caregivers could use the data rather than rely on their care recipients to connect their behaviors to new medical problems that arose over the follow-up period, as Julie (50y, F, 18 m interview) explained, “If we ever saw him going to the bathroom a lot we’d know that he might have some sort of bladder infection or something like that, so that he might not be able to communicate to us.”

Participants could also monitor the change in their care recipient’s habits following medical intervention. Randy, the 74-year-old male who brought the sleeping issues to their physician was able to see that their care recipient, who had been diagnosed with sleep apnea, “sleeps much better with the aid of a CPAP machine. Has not had to get up in the middle of the [night]” (6-month checklist). Amy (58y, F, 18 m interview) was able to monitor improvement in their care recipient’s sleep after they changed medication: “And as a result of being on [the RAM], we could see that the medication allowed her to sleep for longer periods. Prior to that she was up and down every night.” Participants used RAM data to validate their concerns to medical professionals and observe changes in their care recipients’ behavior before and after medical treatment across the 18-month follow-up period.

#### Shifting life contexts

Changing life situations tended to translate to less need for the RAM system. Shifting life contexts often focused on more care being offered by persons in the care recipients’ lives. For instance, Betty (53y, F, 12 m checklist) shared that her mother is “now in assisted living, so we are using [the RAM] less since they are more involved.” Likewise, Terry (60y, F, 6 m checklist) indicated that they “had live-in help all summer” and had not set-up any alerts, “because our son is living with us now.” Terry (12 m checklist) might have needed the RAM more when their son moved out, but “I was laid-off from my job in September 2016, and I currently work part-time from home, so I am caregiving almost 100% of the time now. So, I have not used [the RAM] at all.” This trend continued through 18 months. Similarly, Debra (54y, F, 18 m interview) reported less time needed for care after stepping away from their job: “But after I had to retire and everything, I mean we’re almost pretty much together. You know. And I pretty much watch him closely.”

One exception to these patterns was when Dorothy (88y, F, 18 m interview) experienced a disruptive health event of their own and was not able to rely on the RAM at that crucial time, “We’d go up to the lake three, four days at a time. We’d go out [doing] other things. I had a heart attack. I was in the hospital for six days. There was never any kind of reaction from that system. We never heard anything, never called, never anything.” In a critical instance of needing the RAM, it did not react and subsequently led to diminished confidence in and use of the RAM.

#### Gaining comfort and trust

Caregivers described that their comfort level and trust in the system was only achieved after long-term use and familiarity with the sensors and function of the system. Initially caregivers reported some challenges in explaining the system to the care recipient and family: “And I remember one time after I set it up I went there and she had unplugged it because I think she still didn’t quite grasp what it was doing but now I almost think it’s just more they don’t even really think about it” (Ellen, 59y, F, 18 m interview). In the beginning, caregivers reported not trusting how the system functioned or whether it was functioning appropriately. To better trust the system’s functioning, Theresa said she initially “kept the notepad open and logged in to watch movement/activity at night. I then felt confident that the system WAS watching while I slept...I just needed to know that it was working at the beginning. I would log into my iPad and check movement etc. It was very accurate, so I relied and trusted the system to alert me of changes” (56y, F, 6 m & 18 m checklists). Caregivers described that over time, in-depth understanding of how the RAM system functioned enabled the caregiver to maximize its use. For example, Theresa noted in her semi-structured interview, “If I was doing bookwork or something I felt like I could still track him and so I just felt more confident that I knew what and where he was... So I think it gave me, like I said, those few minutes that I needed to prevent-- hopefully prevent a disaster.” Trust in how the system worked affected how this caregiver responded to the person with dementia’s behavior. Additionally, trusting the system enabled the person with dementia to live independently and at home for longer than the family had anticipated: “It has also built our confidence to leave our loved one in her home and living independently longer than we might have had we not had the system in place so that we know she is safe” (Amy, 58y, F,18 m checklist). Additionally, reliance and trust in the system over time enabled the person with dementia to live at home in their familiar surroundings and was perceived to delay entry into residential long term care settings.

### Quantitative and mixed methods analysis

#### Unconditional growth models

Model fit statistics for unconditional models are reported in Table 2. For role overload, role captivity, and depressive symptoms, the linear growth model provided an optimal balance between fit and parsimony, so this functional form was retained. For all three outcomes, the unconditional models described an average trajectory starting with a relatively low level of role overload, role captivity, and depressive symptoms with slight increases in each of these outcomes over time (for role overload, I = 7.55, *p* < .001; S = .12, *p* = .08; for role captivity, I = 6.25, *p* < .001; S = .30, *p* < .001; for depressive symptoms, I = 11.03, *p* < .001; S = 1.12, *p* < .001). All coefficients reported are unstandardized, and a Holm correction was applied to account for multiple tests across the conditional models.

**Table 2:** Latent Growth Curve Model Fit Statistics

Model	AIC	BIC	Chi-Square	RMSEA [CI]	CFI	SRMR
Role Overload						
Unconditional intercept-only	2986.286	3005.411	Χ^2^ = 28.110, *df* = 8, *p* < .001	.119 [.073, .168]	.883	.143
Unconditional linear	2981.816	3010.503	Χ^2^ = 17.639, *df* = 5, *p* = .003	.119 [.062, .181]	.926	.095
Unconditional quadratic	2978.536	3019.972	Χ^2^ = 6.359, *df* = 1, *p* = .012	.173 [.065, .311]	.969	.039
Conditional linear	2755.658	2879.652	Χ^2^ = 40.325, *df* = 38, *p* = .368	.019 [.000, .059]	.987	.031
Role Captivity						
Unconditional intercept-only	2688.499	2707.624	Χ^2^ = 38.539, *df* = 8, *p* < .001	.146 [.102, .194]	.850	.162
Unconditional linear	2676.845	2705.532	Χ^2^ = 20.885, *df* = 5, *p* = .001	.133 [.077, .195]	.922	.133
Unconditional quadratic	2665.156	2706.592	Χ^2^ = 1.196, *df* = 1, *p* = .274	.033 [0, .204]	.999	.015
Conditional linear	2525.150	2522.508	Χ^2^ = 48.542, *df* = 38, *p* = .118	.041 [.000, .072]	.945	.036
Depressive Symptoms						
Unconditional intercept-only	4779.114	4798.238	Χ^2^ = 43.931, *df* = 8, *p* < .001	.158 [.115, .206]	.809	.113
Unconditional linear	4758.014	4786.700	Χ^2^ = 16.831, *df* = 5, *p* = .005	.115 [.058, .178]	.937	.082
Unconditional quadratic	4760.003	4801.439	Χ^2^ = 10.820, *df* = 1, *p* = .001	.234 [.123, .369]	.948	.060
Conditional linear	4193.475	4317.470	Χ^2^ = 36.740, *df* = 38, *p* = .528	.000 [.000, .052]	1.00	.019

Note. *AIC* Akaike Information Criterion, *BIC* Bayesian Information Criterion, *RMSEA* Root Mean Square Error of Approximation, *CFI* Comparative Fit Index, *SRMR* Standardized Root Mean Residual

#### Conditional growth models

The conditional growth models (see Table 3) indicated that the RAM treatment had no significant effect on the rate of change in role overload (B = .06, *p =* .90), role captivity (B = .39, *p =* .37), or depression (B = .71, *p =* .62) over time.

**Table 3:** Conditional Latent Growth Curve Models for Role Overload, Role Captivity, and Depressive Symptoms

	Role Overload		Role Captivity		Depression	
	Coefficient	SE	Coefficient	SE	Coefficient	SE
For Intercept						
Intercept	7.36***	0.72	5.91***	0.87	10.68***	3.30
Treatment	− 0.44	1.04	− 0.17	1.23	−5.59	3.97
Male	−1.36*	0.41	−1.24	0.41	−1.09	1.58
Caregiver age	−0.03	0.02	−0.02	0.02	− 0.09	0.06
Care recipient ADL	0.11	0.10	−0.09	0.09	−0.03	0.36
Care recipient IADL	−0.01	0.07	0.07	0.07	−0.10	0.26
Care recipient CI	0.04	0.04	0.04	0.04	0.19	0.13
Difficulty navigating	−0.78	0.72	0.22	0.86	−1.58	3.32
Live together	1.78	0.60	0.62	0.61	4.08	1.98
Tx x difficulty navigating	1.82	1.01	0.69	1.17	5.26	3.94
Tx x live together	−1.64	0.75	−0.55	0.74	−0.71	2.56
For linear slope						
Intercept	−0.21	0.27	−0.11	0.20	1.39	0.62
Treatment	0.04	0.40	0.63	0.36	0.25	1.19
Male	0.17	0.16	0.07	0.18	0.24	0.62
Caregiver age	0.00	0.01	0.00	0.01	0.02	0.02
Care recipient ADL	−0.04	0.04	−0.01	0.03	−0.04	0.11
Care recipient IADL	0.01	0.02	−0.02	0.03	0.08	0.07
Care recipient CI	0.00	0.01	0.00	0.01	−0.02	0.04
Difficulty navigating	0.49	0.27	0.35	0.22	−0.16	0.66
Live together	−0.33	0.24	−0.01	0.23	−0.42	0.70
Tx x difficulty navigating	−0.11	0.39	−0.49	0.37	0.71	1.20
Tx x live together	0.28	0.28	−0.09	0.29	−1.02	0.89

Note. *ADL* activities of daily living, *IADL* instrumental activities of daily living, *CI* cognitive impairment

^*^centered to mean

^***^p < .001. ^**^p < .01. ^*^p < .05

Although the qualitative data indicated several dynamic changes in context and situation, many of these domains were not specifically measured in the close-ended survey items. Moderators initially identified in our 6-month analysis as well as the complete longitudinal qualitative analysis here were included (difficulty navigating the home; caregiver lives with care recipient)) [26]. However, no significant interactions were found between the RAM treatment and any of the covariates that emerged as potential moderators of interest from the qualitative findings or our prior 6-month analysis of effectiveness (see Table 3).

## Discussion

One of the benefits of mixed methods designs is that the combined data “strands” yield greater insights than if researchers rely on traditional, empirically driven quantitative results [32]. As is evident in the 18-month randomized controlled evaluation, individual growth curve models did not identify significant nor moderation effects of the RAM technology on any dementia caregiver outcome studied. From the standpoint of the empirical findings, it appeared RAM did not exert statistically significant, nor meaningful, effects on key dementia caregiver outcomes.

Limiting one’s interpretation of the findings to the quantitative results, however, masks the considerable variability in response that was evident in the qualitative findings study component. Specifically, the 18-month time frame used in this evaluation allowed for a greater understanding of how dementia caregivers phased “in and out” of RAM use based on their needs, the contextual complexities of at-home care, and the trajectory of their relatives’ dementia. The diversity of these contextual and temporal dynamics led to dementia caregivers’ perceptions of the system as useful at some time points, but not others. Overall, the qualitative results suggest a complex picture of how RAM technology is utilized and eventually integrated into the routine, day to day care of people living with dementia at home. Effective RAM technology adoption occurred and was bounded by time (e.g., changing perceptions of technology usefulness as a relative’s ADRD progressed), variable physical environments, readiness for technology use, and needs for ongoing technical assistance from a human being. The complexities in context and time indicate a need for alternative design approaches that better capture the varied interplay of RAM technology use, timing, and adoption at the individual and context of care levels.

Although there exist few randomized controlled trials of RAM technology in dementia that feature as long a follow-up as in this study, our findings are similar to another recent, high quality randomized controlled evaluation of RAM and telecare tailored for people with dementia and their caregivers over a 24 week period (the Assistive Technology and Telecare to maintain Independent Living At home for people with dementia/ATTILA trial) [47–49]. Nonetheless, it is important to contrast the demonstration design of the current analysis. Other RAM technology evaluations tend to incorporate more intensive “intervention” approaches that feature a care manager or other professional that receives and interprets monitoring data for the purposes of delivering and enacting necessary care [25]. Our study chose to adopt a different strategy that better reflected how caregivers would routinely use RAM technology. Instead of introducing more intensive professional support, families adapted and used the technology as they saw fit in their own homes. Such an approach maximizes external validity of the findings, but at the same time may have attenuated the potential benefits of the intervention for dementia caregivers. Such a dynamic was apparent in the qualitative findings as well as the process data presented in our prior work [33] which suggested that many dementia caregivers either turned off the sensors or simply did not respond to the alerts to change their day-to-day care.

These findings emphasize the need of professional involvement when using RAM for people with dementia and their caregivers at home. Unlike residential care environments, professionals may not need to receive, interpret, and enact upon all RAM alert data for people living with dementia at home. However people with dementia and their caregivers who live at home may requre more intensive engagement in not just installing and reviewing RAM but also adapting the system on an ongoing basis to tailor the placement of sensors, the information generated, and other key information that are deemed useful for people living with dementia and their caregivers. As our qualitative themes here indicate, the earlier phases of RAM adoption appear particularly critical for dementia caregivers in the community. Such engagement likely requires more than periodic “check in” calls or contacts, but active oversight and review of how dementia caregivers utilize technology such as RAM to ensure the technology is appropriate and beneficial for their particular care contexts.

A risk of technology innovations is that unless they are seamlessly integrated into day-to-day care routines and can flexibly address a range of needs (rather than very specific ones) for people with ADRD and/or their caregivers, the technology introduced simply becomes another stressor. This was evident again in the qualitative findings and themes, where the fit of the technology waxed and waned over the course of the evaluation. Periods of adjustment and ongoing support appeared linked to greater acceptance of RAM on the part of caregivers as well as care recipients with dementia. This heterogeneity of responses/experiences utilizing RAM technology was not evident in the empirical randomized controlled evaluation results.

Our preliminary, 6-month evaluation of the RAM technology on dementia caregiver outcomes suggested the presence of a moderational effect. Specifically, dementia caregivers who assisted relatives with less severe cognitive impairment or had difficulty navigating around the home environment indicated statistically significant increases in competence and self-efficacy, respectively [26] However, similar patterns of results were not evident in this larger, longer randomized controlled evaluation. This was due to the analytic approach selected for the current analysis: neither self-efficacy nor competence demonstrated sufficient statistical variability over the 1.5-year period to consider their inclusion in the conditional growth curve models. For these reasons, we did not have the opportunity to determine whether severity of cognitive impairment or difficulty navigating the home similarly moderated RAM technology’s effects of dementia caregivers’ competence or efficacy over time.

There exist several barriers to technology adoption among older persons and their caregivers. Barriers that may influence whether and how people living with dementia and their caregivers successfully utilize technology include access and interest; mismatch in age-related changes in cognition or other abilities that may adversely influence one’s ability to utilize technology that is not necessarily designed for use by older persons or family caregivers; concerns about privacy (which are particularly relevant in the context of unobtrusive monitoring technologies such as RAM); and the increasing specialization of technologies designed for certain tasks but not created to address multiple caregiving needs simultaneously [5, 50, 51]. The latter is a particular concern as technologies to support at-home care continue to proliferate. If caregivers must repeatedly learn to use specific technologies that only address limited care needs, there is increased risk that the technology is viewed less as a solution and instead as an additional care-related stressor.

There are numerous technologies that now exist both in the general marketplace as well as in the research literature that purport to address the health and functional needs of older persons and their caregivers. Unlike many other studies of technology that largely focus on feasibility or acceptability, the current study subjected RAM to a randomized controlled evaluation and thus could be considered a step forward in terms of contributing to the scientific evidence. Moreover, the findings, particularly when the qualitative and quantitative study results are considered, suggest important practice implications. Specifically, the findings illustrate an important clinical gap: the need to develop a systematic process to match dementia caregiver needs with available technologies (e.g., see [47]). Currently caregivers and health professionals are often left to make decisions to adopt certain technologies with little information. Moreover, those who recommend technologies may not fully grasp the scope of the dementia caregiving context and may be unaware of the full capabilities or limitations of a particular technology. Such issues are magnified when a commercial imperative is introduced, as targeting and matching the right technologies with specific caregiving situations may not align with the profit considerations of some technology developers where mass purchase is most desirable. Nonetheless, developing a validated tool/process to match dementia caregiver needs with an appropriate technology would likely result in more promising (and likely person-centered) outcomes for dementia caregivers [47].

In addition to the concerns above, there were other limitations. The sample was volunteer based, largely White, and not representative of the dementia caregiver population in the U.S., and although some rural caregivers were included there is a significant scientific and clinical need to understand how RAM or other types of technologies are appropriate for people and families who are black, indigenous, and persons of color [52]. Researchers’ blinding to treatment assignment did not occur due to available resources and staffing at the outset of the project. There was some loss to follow-up in our sample; loss to follow up was 5.6% at 6 months, 9.5% at 12 months, and 11.7% at 18 months. Several of the themes and responses from the qualitative component suggested additional key variables/domains that, if measured, may have had statistically significant and relevant effects in the quantitative component (i.e., comfort with the RAM technology). However, these measures were not available in the close-ended surveys. In contrast to the design used here, a mixed methods design that adopted more of a multi-phase, sequential approach (a detailed qualitative study that informed measurement selection, followed by a randomized controlled evaluation of RAM technology for people with dementia and their caregivers that incorporated measures identified in the qualitative component) may have yielded more consistent empirical findings.

## Conclusions

This study builds upon and extends prior evaluations of RAM or similar unobtrusive technologies for people with dementia and their caregivers. The lack of quantitative effects did not conform to our expectations [48, 49], but the use of mixed methods yielded considerable insights as to why statistical effects did not occur. The findings also provide intriguing pathways for future research on RAM or similar technologies in the dementia care context. Aligning evaluation methodology (e.g., more advanced intervention designs that incorporate re-randomization for those dementia caregivers who demonstrate comfort with RAM) that is parallel to highly individualized, tailored technologies is likely necessary to determine how and under what conditions RAM or similar technologies exert benefits for people living with dementia at home as well as their family caregivers.

## Supplementary Information

**Additional file 1.** Semi-Structured Interview Guide: Post-Randomized Controlled Evaluation Embedded Component.

## Data Availability

The datasets generated and/or analyzed during the current study are not publicly available due to the first author’s/principal investigator’s decision to make the data publicly available upon completion of primary outcome analyses. When ready, the datasets generated and analyzed in the current study will be made available on the National Archive of Computerized Data on Ageing as well as the University of Minnesota Data Repository for U of M.

## Abbreviations

ADLs: Activities of daily living
ADRD: Alzheimer’s disease or related dementias
AIC: Akaike Information Criterion
BIC: Bayesian Information Criteria
DHT: Director of Health Technology
F: Female
IADLs: Instrumental activities of daily living
LTSS: Long-term services and supports
M: Male
m: Month
RAM: Remote activity monitoring
RMSEA: Root mean square error of approximation
SD: Standard deviation
SLUMS: St. Louis University Mental Status examination
SPM: Stress Process Model
SSCQ: Short Sense of Competence Questionnaire
y: Years old
