# Supplementary material for: Remote activity monitoring for family caregivers of persons living with dementia: a mixed methods, randomized controlled evaluation
Source: BMC Geriatr. 2021 Dec 18;21:715. doi: 10.1186/s12877-021-02634-8 (PMC8684277; doi:10.1186/s12877-021-02634-8)
Supplement: Supplementary file 1 — Additional file 1. Semi-Structured Interview Guide: Post-Randomized Controlled Evaluation Embedded Component. [file 12877_2021_2634_MOESM1_ESM.doc]

**Semi-Structured Interview Guide: Post-Randomized Controlled Evaluation Embedded Component**

| Introduction  Thank you so much for participating in our evaluation of the eNeighbor remote sensor technology. To follow-up on your participation, I would like to ask some open-ended questions. My objective in this interview is to determine why eNeighbor benefited you and the person with memory loss or not. |
| --- |
| Opening Question   1. Just to get us started, I’d like each of you to tell me a little bit more about the person with memory loss. How did your first find out about her/his memory problems?   Probes: When did you first start providing help to the person with memory loss because of her/his dementia? What are things like now? |
| Introductory Question  2. Why did you decide to enroll in this project and use eNeighbor? |
| Benefits  3. What were some of the benefits of eNeighbor to you? Why?  4. What were some of the benefits of eNeighbor to the person with memory loss? Why?  5. [How did you think eNeighbor helped you?]  6. Why do you think eNeighbor helped you? Why not?  7. [How did you think eNeighbor helped the person with memory loss?  8. Why do you think eNeighbor helped the person with memory loss? Why not? |
| Harm and drawbacks  9. Do you believe that eNeighbor resulted in any negative outcomes for you or the person with memory loss? Which ones? [How and why] do you think this happened? |
| eNeighbor components  10. I’d like to walk through the various service components of eNeighbor. I’d like you to describe [how] each component did or did not help you or the person with memory loss, and why:  10a. The needs assessment the Director of Nursing and Technology completed with you and the person with memory loss.  10b. The remote sensors and the alerts they generated.  10c. The care plan that the Director of Nursing and Technology developed with you.  10d. The ongoing monitoring and assistance of the Director of Nursing and Technology.  10e. The myHealthsense portal. |
| Links to outcomes  11. [How do] you think the use of eNeighbor had any effect on the following? Why or why not?  11a. Your feelings of confidence and capability in providing care to  the person with memory loss.  11b. Your feelings of stress, fatigue, and being overwhelmed as a  caregiver.  11c. Preventing health crises on the part of the person with memory loss, such  as wandering or falls.  11d. Helping the person with memory loss stay at home as long as possible.  11e. Help you and the person with memory loss avoid unnecessary hospital or  emergency room visits. |
| Ending Question  10. Thank you for helping us learn more about your experiences with  eNeighbor. Is there anything else that you would like to say [about how and why eNeighbor  worked for you and the person with memory loss or not?] |
